# Novel Ti–Zr–Hf–Fe Nanostructured Alloy for Biomedical Applications

**DOI:** 10.3390/ma6114930

**Published:** 2013-10-25

**Authors:** Anna Hynowska, Andreu Blanquer, Eva Pellicer, Jordina Fornell, Santiago Suriñach, Maria Dolors Baró, Sergio González, Elena Ibáñez, Lleonard Barrios, Carme Nogués, Jordi Sort

**Affiliations:** 1 Departament de Física, Facultat de Ciències, Universitat Autònoma de Barcelona, E-08193 Bellaterra, Spain; E-Mails: jordinafornell@gmail.com (J.F.); santiago.surinyach@uab.cat (S.S.); dolors.baro@uab.es (M.D.B); sergiogs10@yahoo.es (S.G.); 2 Departament de Biologia Cellular, Fisiologia i Immunologia, Universitat Autònoma de Barcelona, E-08193 Bellaterra, Spain; E-Mails: andreublanquer@gmail.com (A.B.); elena.ibanez@uab.cat (E.I.); lleonard.barrios@uab.cat (L.B.); 3 Institució Catalana de Recerca i Estudis Avançats (ICREA) and Departament de Física, Universitat Autònoma de Barcelona, E-08193 Bellaterra, Spain; E-Mail: jordi.sort@uab.es

**Keywords:** Ti-based alloy, biomaterial, microstructure, mechanical behavior, corrosion performance

## Abstract

The synthesis and characterization of Ti_40_Zr_20_Hf_20_Fe_20_ (atom %) alloy, in the form of rods (ϕ = 2 mm), prepared by arc-melting, and subsequent Cu mold suction casting, is presented. The microstructure, mechanical and corrosion properties, as well as *in vitro* biocompatibility of this alloy, are investigated. This material consists of a mixture of several nanocrystalline phases. It exhibits excellent mechanical behavior, dominated by high strength and relatively low Young’s modulus, and also good corrosion resistance, as evidenced by the passive behavior in a wide potential window and the low corrosion current densities values. In terms of biocompatibility, this alloy is not cytotoxic and preosteoblast cells can easily adhere onto its surface and differentiate into osteoblasts.

## 1. Introduction

Materials for permanent biomedical implants must be designed in order to guarantee a long lifetime (longevity) of the implant after being inserted into the human body. Metallic alloys present an immense potential for orthopaedic applications, due to their excellent mechanical strength and resilience, which is outstanding in comparison with polymers or ceramics [1]. Permanent implants should contain neither toxic nor allergic elements (e.g., Al, V, Ni, Co) and should possess high corrosion and wear resistances. A good structural and functional connection between living bone and the surface of a load-bearing artificial implant is also essential.

In recent years, Ti-based alloys have gathered special attention in the biomedical field as they show a combination of high strength, reduced stiffness, low density, good biocompatibility, and good corrosion resistance, in many cases superior to conventional steel or Co–Cr alloys [2]. Pure Ti and Ti–6Al–4V (composed of a mixture of α + β phases) alloys are currently the most widely used structural biomaterials for the replacement of hard tissues in artificial joints. Although pure titanium has acceptable mechanical properties, in most applications Ti is alloyed with small amounts of aluminium and vanadium, with the aim of stabilizing the β-phase, which exhibits lower Young’s modulus than the α-phase. However, the release of V and Al ions to the human body causes long-term health problems, such as peripheral neuropathy, osteomalacia, and Alzheimer diseases [3]. Thus, new Ti-based alloys with low Young’s modulus (to ensure good mechanical compatibility with bone) and free from non-biocompatible elements (e.g., Al, V, Ni, or Co), are currently required for the next generation of metallic implant materials.

Besides the alloy composition, the microstructure (*i.e*., grain size and amounts of interphase boundaries) also plays a key role in the resulting physical and chemical properties of synthesized materials. Previously, BMG (bulk metallic glasses) were developed and found to be good candidates fulfilling the requirements of the orthopaedic field. The advancement of BMG led to the discovery of a new nanostructured alloy, which may exhibit even more outstanding properties than BMG and conventional Ti alloys. These nanostructured materials have attracted much interest in the last decade due to their size-dependent (a grain size typically lower than 100 nm) unique mechanical, physical and chemical properties [4]. They can be prepared by a number of different techniques (ball milling and subsequent hot pressing, severe plastic deformation, suction casting, *etc.*) and the grain size, morphology and composition can be controlled by tuning the processing parameters. In comparison to coarse-grained materials, nanocrystalline alloys show higher strength and hardness at room temperature [5].

Currently, a number of multi-component Ti-based alloys showing nanocrystalline structure and not containing the highly-toxic (Be, Ni, Al, V, Co, Cr, *etc.*), such as Ti–50Ta [6], Ti–Sn–Nb [7], Ti–Mo–Nb [8], Ti–Mo–Zr–Fe, Ti–Nb–Ta–Zr [9,10], or Ti–Fe–Sn [11], are of special interest for the biomedical field. These alloys constitute so-called second generation biomaterials [1,3,12].

In this paper we focus our attention on the Ti_40_Zr_20_Hf_20_Fe_20_ (atom %) alloy. Samples with this composition had already been prepared but only in ribbon shape (a few tens of μm in thickness) and a mixture of amorphous plus nanoscale metastable icosahedral phase, with an average size of 5 nm, was obtained [13]. It was suggested that Fe, due to its high glass-forming ability, could trigger the formation of the amorphous phase also in bulk form or, at least, promote a nanocrystalline structure [13]. Hence, a decrease of the Young’s modulus is anticipated in both scenarios. On the one hand, amorphous materials typically exhibit lower Young’s modulus than their crystalline counterparts (a behavior termed “elastic softening”) [14,15]. On the other hand, Fe is a β stabilizer element, therefore in Fe-containing nanocrystalline alloys one can expect a lowering of the Young’s modulus and a concomitant increase in hardness [16,17,18], Moreover, Fe is considered as a suitable element for biodegradable implants and it has been demonstrated to be as high as 80% viable after four days in culture with L-929 of [19]. Likewise, the addition of Zr and Hf elements, which belong to the same family as Ti in the periodic table, presumably leads to enhanced mechanical properties, good corrosion resistance and good biocompatibility [20]. The Ti_40_Zr_20_Hf_20_Fe_20_ alloy is prepared in the form of rods of 2 mm in diameter. The mechanical and electrochemical corrosion properties are assessed and compared to the commercial Ti–6Al–4V alloy. Standardized *in vitro* cytotoxicity assays to evaluate the biocompatibility are also performed. The results reveal the excellent performance of Ti–Zr–Hf–Fe, making this alloy a potential candidate to be used in the biomaterial field.

## 2. Results and Discussion

### 2.1. Microstructure

**Figure 1:**
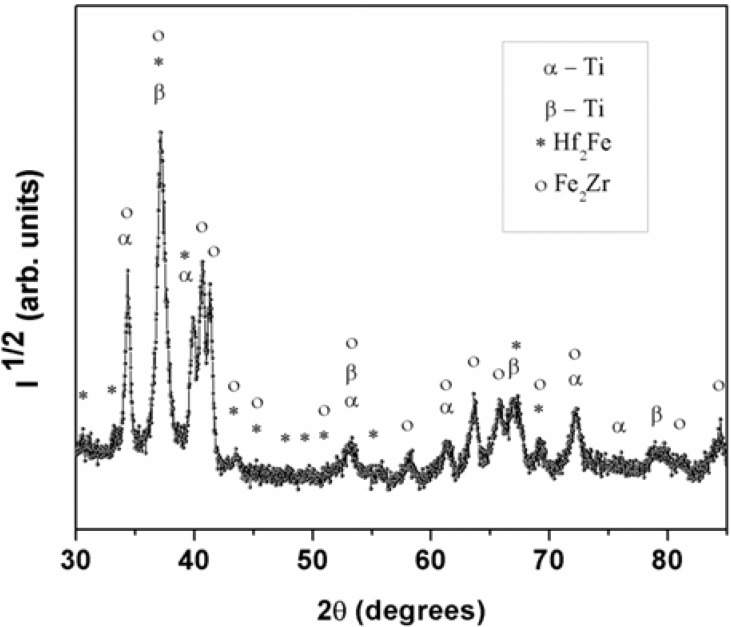
XRD pattern corresponding to the as–cast Ti_40_Zr_20_Hf_20_Fe_20_ alloy.

Figure 1 shows the XRD pattern of the Ti_40_Zr_20_Hf_20_Fe_20_ as-cast sample. A fully crystalline structure is observed, as noted from the absence of broad halos, typical of amorphous materials. Based on the databases (X’Pert HighScore, FindIt, PCPDFWIN programs), the main reflections of the XRD pattern are ascribed to cubic Hf_2_Fe (space group Fd3m, *a* = 11,77 Å) and hexagonal Fe_2_Zr (space group P6_3_/mmc, *a* = 5.23 Å; *c* = 8.37 Å) intermetallic phases as well as to hexagonal α-Ti (space group P6_3_/mmc, *a* = 3.01 Å; *c* = 4.53 Å), and cubic β-Ti (space group Im3m, *a* = 3.42 Å) phases, in agreement with previous works [13]. The diffraction peaks are slightly shifted from the tabulated ones. This is usually observed in multicomponent systems where atoms of different species can act as substitutional atoms. For instance the theoretical cell parameter for β-Ti phase is *a*_th._ = 3.31 Å which is slightly smaller than the experimental one (*a*_expt._ = 3.42 Å). This can be explained by the larger atomic size of Zr and Hf which causes an increment in the lattice parameters.

**Figure 2:**
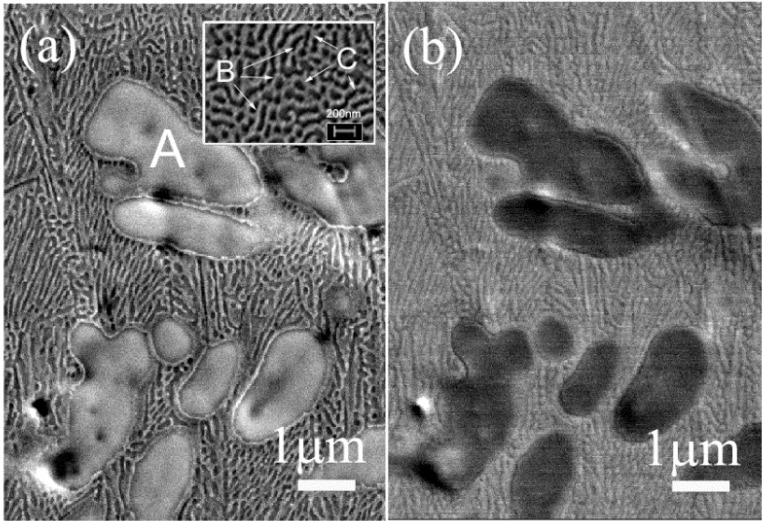
SEM images of the Ti_40_Zr_20_Hf_20_Fe_20_ alloy taken from the central part of the cross-section area of the rod. Panel (**a**) is a secondary electrons image and (**b**) a backscattered electrons image, both showing the coexisting of different zones (A, B, and C). Zone A corresponds to large size dendrites, while zones B, C belong to the small lamellae composing the eutectic matrix (2a inset). The size of the inset in (**a**) is a zoom of 1.2 μm × 0.9 μm.

Figure 2 shows secondary (a), and backscattered (b), electron images corresponding to the central part of the cross-section of the rod. Remarkably, a similar microstructure was noticed at the edges of the rods. These materials exhibit a composite-like microstructure, with the presence of at least three different regions that display different brightness (zones A, B, and C). Zone A is assigned to micrometer-sized dendrites as shown in Figure 2a, while zones B and C (Figure 2a) are the alternated bright and dark structure of the eutectic matrix composed of nanoscale lamellae (width around 100 nm). The varying lamellae geometry is due to different orientations of the eutectic colonies. Hence, upon rapid quenching, the multi-component Ti_40_Zr_20_Hf_20_Fe_20_ liquid underwent an eutectic reaction, forming, *in situ*, a composite material made of micrometer-sized dendritic (Ti-based solid solution) regions surrounded by an ultra-fine grained eutectic matrix. This kind of microstructure should promote a high strength, in comparison to commercial Ti–6Al–4V alloy [21].

**Figure 3:**
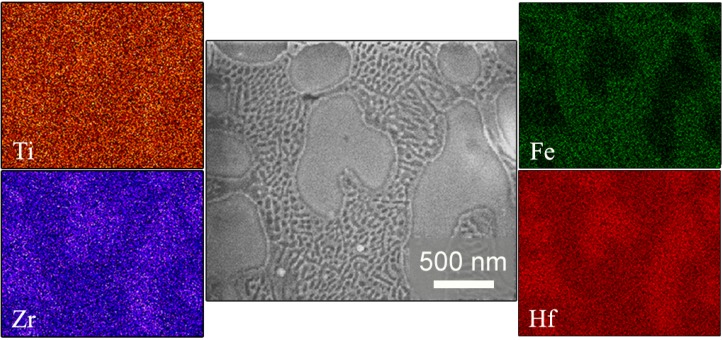
SEM image of a central region of the Ti_40_Zr_20_Hf_20_Fe_20_ sample, together with the compositional energy dispersive X-ray mappings corresponding to Ti, Zr, Fe, and Hf elements.

Energy dispersive X-ray (EDX) analysis (Figure 3) indicates that Zr and Hf are in a larger amount inside the dendrites, whereas Fe is mainly located in the eutectic matrix. Ti is distributed equally along the selected area of the transverse cross-section of the surface. Based on the compositional analyses and the XRD results, α-Ti and β-Ti with some additional elements in solid solution (partial substitution of Ti for Hf or Zr) could be present in both the dendrites and the eutectic matrix. Fe_2_Zr and Hf_2_Fe phases are likely to be located in the eutectic matrix, since more Fe is detected in this region.

### 2.2. Mechanical Properties

Table 1 lists the values of the Poisson’s coefficient (*v*), Young’s modulus (*E*), shear modulus (*G*), and bulk modulus (*K*). The values of Young’s and shear modulus for the Ti_40_Zr_20_Hf_20_Fe_20_ are lower than for the commercial Ti–6Al–4V. On the contrary, the values of Poisson’s coefficient and bulk modulus are slightly higher compared to the values of commercial alloy.

**Table 1:** Summary of the elastic properties (*v*, *E*_Acoust_, *G* and *K* denote the Poisson’s coefficient, Young’s modulus, shear modulus and bulk modulus, respectively) of the as-cast Ti_40_Zr_20_Hf_20_Fe_20_ alloy. Results for the commercial Ti–6Al–4V are shown for comparison purposes.

Sample	*v*	*E*_Acoust_ (GPa)	*G* (GPa)	*K* (GPa)
Ti_40_Zr_20_Hf_20_Fe_20_	0.359 ± 0.001	102.2 ± 0.3	37.6 ± 0.3	132.3 ± 1.7
Ti–6Al–4V	0.326 ± 0.003	111.5 ± 1.1	42.0 ± 0.4	106.6 ± 1.1

**Figure 4:**
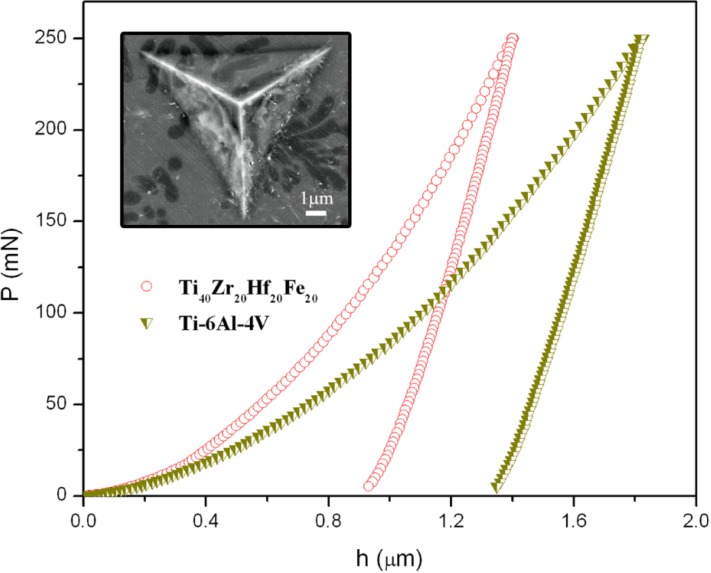
Load-displacement (*P–h*) nanoindentation curves of the Ti–6Al–4V and Ti_40_Zr_20_Hf_20_Fe_20_ alloys obtained applying a maximum force *P*_Max_ = 250 mN. The inset is a backscattered SEM image showing an indent made close to the centre of the Ti_40_Zr_20_Hf_20_Fe_20_ sample (*P*_Max_ = 250 mN).

Figure 4 shows representative nanoidentation load-displacement (*P*–*h*) curves of the Ti_40_Zr_20_Hf_20_Fe_20_ and commercial Ti–6Al–4V alloys, measured up to a maximum load *P*_Max_ = 250 mN. Indentations using such a high load are large enough to embrace all the existing phases (as shown in the inset of Figure 4).

**Figure 5:**
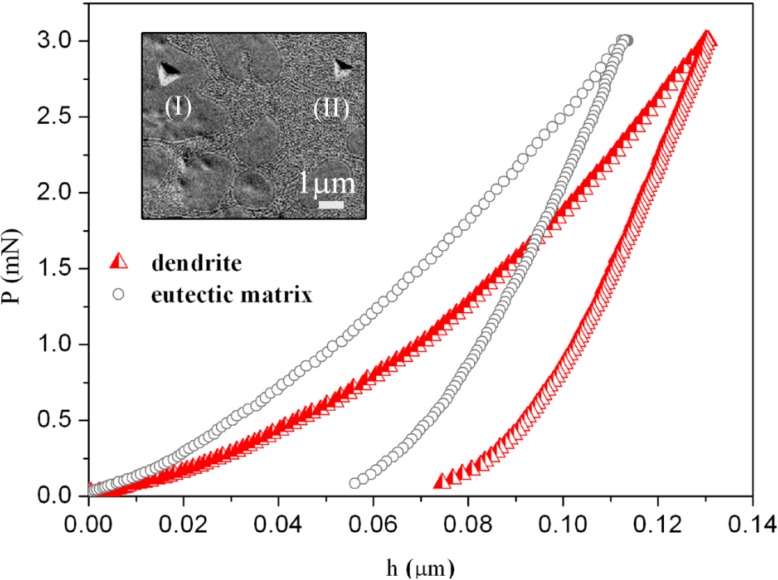
Load-displacement (*P–h*) nanoindentation curves for Ti_40_Zr_20_Hf_20_Fe_20_ alloy, corresponding to a dendrite (red curve) and eutectic matrix (grey curve). Shown in the inset is a backscattered SEM image of the indents: (**I**) inside the dendrite and (**II**) inside the eutectic matrix (*P*_Max_ = 3 mN).

For the Ti_40_Zr_20_Hf_20_Fe_20_ alloy, nanoindentation tests using *P*_Max_ = 3 mN were also performed in order to determine the mechanical properties of the dendrites and the eutectic matrix (Figure 5). The values of hardness (*H*), reduced Young’s modulus (*E*_r_), *H*/*E*_r_, *H*^3^/*E*^2^_r_ ratios (related to the wear resistance), as well as the ratios *U*_el_/*U*_tot_ and *U*_pl_/*U*_tot_ (where *U*_tot_ = *U*_el_ + *U*_pl_) for maximum applied load of 250 mN, are listed in Table 2.

**Table 2:** Summary of the values of hardness (*H*), reduced Young’s modulus (*E*_r_), *H*/*E_r_*, *H*^3^/*E*^2^_r_, *U*_el_/*U*_tot_, and *U*_pl_/*U*_tot_ (where *U*_el_, *U*_pl_ and *U*_tot_ denote the elastic, plastic and total indentation energies, respectively), corresponding to the Ti–6Al–4V and Ti_40_Zr_20_Hf_20_Fe_20_ alloys extracted from the nanoindentation curves performed with maximum load of 250 mN.

Sample	*H* (GPa)	*E*_r_ (GPa)	*H*/*E*_r_	*H*^3^/*E*r^2^ (GPa)	*U*_el_/*U*_tot_	*U*_pl_/*U*_tot_
Ti_40_Zr_20_Hf_20_Fe_20_	8.7 ± 0.2	104 ± 3	0.083 ± 0.003	0.060 ± 0.006	0.554 ± 0.019	0.446 ± 0.016
Ti–6Al–4V	5.0 ± 0.1	121 ± 3	0.041 ± 0.001	0.009 ± 0.001	0.254 ± 0.005	0.746 ± 0.014

The Ti_40_Zr_20_Hf_20_Fe_20_ rod-shape alloy is mechanically harder than the Ti–6Al–4V one, as can be deduced from *(P–h)* curve from the larger values of penetration depth of Ti–6Al–4V [22]. The values of hardness of dendrite and eutectic matrix determined using applied load of 3mN are equal to 7.5 GPa and 10.5 GPa, respectively (Figure 5). The average hardness from low-load indentation tests is 9.3 GPa. This is the value calculated for all nanoindentation tests (*i.e.*, from the values of hardness obtained in the dendrites, eutectic matrix and from indentations at the border between dendrites and eutectic regions) (~100 tests). The difference between the hardness at 3 mN (*H* = 9.3 GPa) and 250 mN (*H* = 8.7 GPa) is likely due to the indentation size effect (ISE) [23]. The ISE has been ascribed to a variety of factors, such as surface effects [24], friction between the indenter and the sample [25], or, more recently, strain-gradient hardening [26,27]. This latter considers that, as a result of the shear field created by the indenter, the crystal lattice becomes distorted and (extra) geometrically necessary dislocations, besides the statistically stored dislocations, have to be created to account for the large shear strains. For large indentations, the strain variation between two extremes is more gradual and the statistically stored dislocations can easily accommodate the shear stress without the need of geometrically necessary dislocations, thus, reducing strain-gradient effects. The eutectic structure is mechanically harder than the dendrites probably because of the smaller grain size and the presence of the intermetallic phases [21,28]. Namely, the large amounts of inter-phase boundaries existing in the eutectic regions hinder dislocation motion and result in increase of strength, an effect known as Hall-Petch [29,30]. The magnitude of the observed strengthening depends upon structure of the grain boundaries and the degree of *misorientation* between grains. The higher the applied stress is needed to propagate dislocation, the larger is the resulting hardness value [31,32].

Concerning the reduced Young’s modulus, the studied Ti_40_Zr_20_Hf_20_Fe_20_ alloy shows a lower value (104 GPa) than the Ti–6Al–4V alloy (121 GPa), in agreement with acoustic measurements. The difference can be explained by both the relative amount of β phase (*i.e*., their distinct microstructure) and the chemical composition. On the one hand, the amount of β phase in the Ti_40_Zr_20_Hf_20_Fe_20_ is higher than in the commercial Ti–6Al–4V [22]. On the other hand, the presence of Hf would lead to a decrease of the Young’s modulus. It has been demonstrated that the Young’s modulus in quenched Ti–Hf alloys with similar microstructure slightly decreases with an increase in the Hf content [33]. Remarkably, the value of *E*_r_ for Ti_40_Zr_20_Hf_20_Fe_20_ is similar to that of Ti_40_Cu_38_Zr_10_Pd_12_ bulk metallic glass (BMG) [34]. In addition, listed in Table 2 are the ratios *H*/*E*_r_ and *H*^3^/*E*_r_^2^ for both alloys. These parameters are related to wear resistance and are important to assess the lifetime of the implant. *H*/*E*_r_ stands for the elastic strain to failure [35], while *H*^3^/*E*_r_^2^ is related to the resistance of a material to plastic deformation in loaded contact. Due to strong hardness and relatively low Young’s modulus of Ti_40_Zr_20_Hf_20_Fe_20_, the values of *H*/*E*_r_ and *H*^3^/*E*_r_^2^ are larger than for Ti–6Al–4V. Interestingly, the elastic recovery, *U*_el_/*U*_tot_, is also higher in the new Ti_40_Zr_20_Hf_20_Fe_20_ alloy, hence, this material would be more resistant to impact loading than Ti–6Al–4V.

### 2.3. Corrosion Behavior

As aforementioned, a material intended for implant applications should be free from toxic and allergenic elements and should not be extensively affected by pitting corrosion. For this purpose, interest is laid on designing suitable alloy compositions, microstructures (and crystallite sizes) that are immune in a body fluid environment. Smaller crystallite size typically leads to poorer corrosion performance due to the larger volume fraction of interfaces [36]. The presence of a passive surface layer (typically oxides) on most of the metallic alloys presently used as implants precludes their dissolution when in contact with bodily fluids. The resistance of the passive layer toward the corrosion attack depends on both the alloying elements of the base material and the nature of oxides formed. Ti alloys are widely used in orthopaedic and dental fields because of their combination of excellent corrosion resistance and unique biocompatibility.

**Figure 6:**
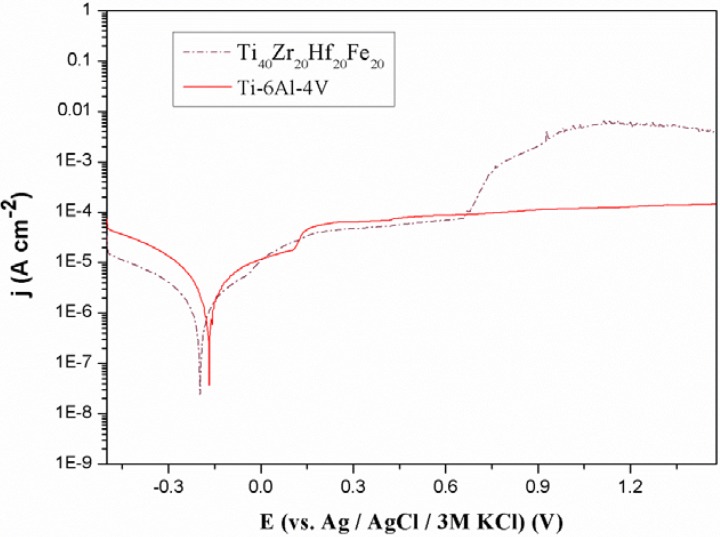
Polarization curves of the as-cast Ti_40_Zr_20_Hf_20_Fe_20_ and commercial Ti–6Al–4V alloys in naturally aerated Hank’s solution at 37.5 °C.

Figure 6 shows representative potentiodynamic polarization curves of the Ti_40_Zr_20_Hf_20_Fe_20_ and Ti–6Al–4V alloys. The corresponding corrosion potential, *E*_corr_, the corrosion current density, *j*_corr_, and the polarization resistance, *R*_p_, values are listed in Table 3.

**Table 3:** Summary of the electrochemical parameters obtained for the studied Ti_40_Zr_20_Hf_20_Fe_20_ and commercial Ti–6Al–4V alloys. The values of *j*_corr_, *E*_corr_, *E*_pit_, and *R*_corr_ denote the corrosion density, the corrosion potential and polarization resistance.

Sample	*j*_corr_ (A·cm^−2^)	*E*_corr_ (V)	*R*_corr_ (Ω·cm^2^)
Ti_40_Zr_20_Hf_20_Fe_20_	9.1 × 10^−7^	−0.197	1.4 × 10^4^
Ti–6Al–4V	2.1 × 10^−6^	−0.168	6.9 × 10^3^

Compared to commercial Ti–6Al–4V alloy, the Ti_40_Zr_20_Hf_20_Fe_20_ sample exhibits lower *j*_corr_ and higher *R*_p_ values. However, passivity breakdown is observed at 0.7 V. This can be attributed to both the microstructural features and chemical composition of the Ti_40_Zr_20_Hf_20_Fe_20_ alloy. For example, Fe-containing alloys typically show passivity breakdown in chloride-containing solutions [37]. Nevertheless, such breakdown occurs beyond the limit (0.6 V *versus* Ag/AgCl) typically set for a material to be safely used as a permanent medical implant [38]. Hence, the present alloy still holds promise to be used in the biological field.

### 2.4. Biological Tests

#### 2.4.1. Cell Viability

The potential cytotoxic effect of Ti_40_Zr_20_Fe_20_Hf_20_ alloy on preosteoblast cultures was analyzed using indirect and direct studies at the same time. After 24 h in culture, no significant differences were observed in the percentages of live cells among the three conditions analyzed, that is, cells adhered to the surface of the alloy (direct studies), to the coverslip in presence of the alloy (indirect studies) and to the coverslip in absence of the alloy (control cells). In all cases, more than 97% of preosteoblasts were alive (direct studies 98.2%, indirect studies 97.1% and control cells 98.1%). These results indicate that the alloy has no toxic effect on preosteoblast cell cultures. Our results are in agreement with other studies which indicated that Zr and Hf are nontoxic elements that can be used as alloying elements [39]. Cations released from the alloy after 24 h are not cytotoxic for cells growing in the presence of the alloy.

#### 2.4.2. Cell Adhesion and Morphology

Cell reaction to a material surface is an important factor to be considered. Preosteoblasts are adherent cells which need to attach to a surface to resume cell cycle and proliferate. Cell morphology and focal contacts are indicatives of cell response to a surface. We demonstrate that preosteblasts can adhere to the surface of the alloy indicating that the mirror-like surface of Ti_40_Zr_20_Fe_20_Hf_20_ is an excellent platform for preosteoblasts to attach to and proliferate.

**Figure 7:**
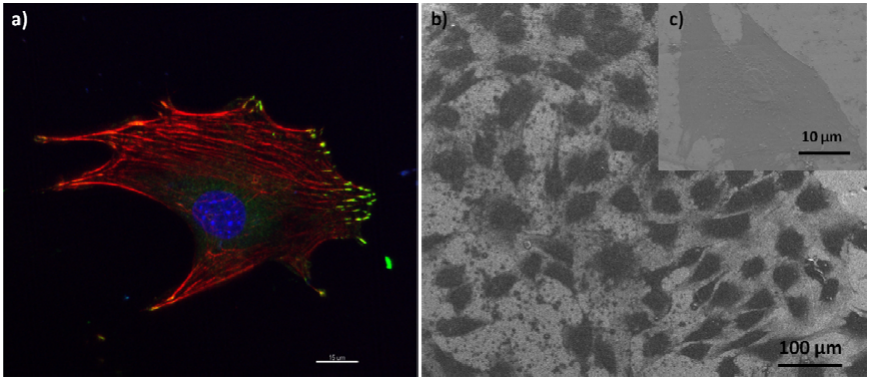
Preosteoblast adhesion to the surface of Ti_40_Zr_20_Fe_20_Hf_20_ alloy: (**a**) Confocal microscopy image of a preosteoblast showing focal contacts (green), stress fibers (red) crossing the cell, and a nucleus (blue); (**b**) Scanning electron microscopy image showing the polygonal morphology of the preostoblasts (enlarged detail in (**c**)).

Figure 7 shows adhesion (a) and morphology (b) of preosteoblasts grown on the top of the alloy. As it can be observed, cells are completely attached to the surface of the alloy by focal contacts at the cell periphery, colocalizing with the extremities of actin filaments (stress fibers), which are well-defined (some of them crossing the cell). Cell adhesion is the first step to occur when cells are seeded on a surface (alloy or culture plate), as adhesion allows to control the behavior of cells and defines their morphology. No differences in the morphology of preosteoblasts adhered onto the Ti_40_Zr_20_Fe_20_Hf_20_ surface and control coverslips were observed. In both cases, cells exhibited similar polygonal shapes with cytoplasmic extensions and with no specific orientation.

#### 2.4.3. Cell Differentiation

**Figure 8:**
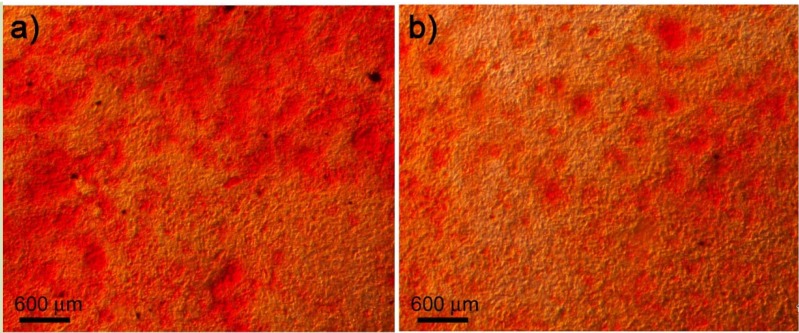
Mineralization of the extracellular matrix by differentiated osteoblasts after 14 days in culture (**a**) in the presence of the alloy and (**b**) without the alloy. Calcium carbonate can be observed using Alizarin Red S staining (red).

For Ti_40_Zr_20_Fe_20_Hf_20_ alloy to be considered a biocompatible material, it must allow cell differentiation to occur. After 14 days in culture with differentiation medium (in presence and absence of the alloy), some nodules of calcium phosphate were detected among the preosteoblasts (Figure 8). After 21 days, a large number of nodules were observed. Extracellular matrix mineralization was similar in osteoblasts in contact with the alloy, in presence of the alloy or in absence of it. Therefore, Ti_40_Zr_20_Fe_20_Hf_20_ alloy allows the differentiation of preostoblasts into osteoblasts.

## 3. Experimental Section

### 3.1. Material and Sample Preparation

Ti_40_Zr_20_Hf_20_Fe_20_ (atom %) master alloy had been prepared by arc melting a mixture of the high purity elements in Ar atmosphere. Subsequently, the master alloy was suction casted into a Cu mold to produce the 2 mm diameter rod.

### 3.2. Structural Characterization

The sample was structurally characterized by X-ray diffraction (XRD), using a Philips X’Pert diffractometer with monochromatic Cu-K radiation. The XRD pattern was acquired using a step-scan mode. The 2 step size was 0.03° and the waiting time per step was selected to be 7 s. A scanning electron microscope (SEM—Zeiss Merlin), equipped with an energy dispersive X-ray (EDX) spectrometer was used for microstructure observation and compositional analysis.

### 3.3. Mechanical Behavior

Nanoindentation experiments were performed on the as-cast sample using a UMIS equipment from Fischer-Cripps laboratories, furnished with a Berkovich pyramidal-shaped indenter tip. The thermal drift was always kept below ±0.05 nm·s^−1^. Arrays of 50 indentations with applied loads of 250 mN and 3 mN were carried out to probe both the average and local mechanical behavior and to verify the accuracy of the indentation data. Prior to the nanoindentation tests, the specimens were carefully polished to mirror-like appearance using diamond paste. The method of Oliver and Pharr was used to determine the hardness and the reduced Young’s modulus [40]. Proper corrections for the contact area (calibrated with a fused quartz specimen), the instrument compliance and initial penetration depth were applied. Finally, the elastic recovery was evaluated as the ratio between the elastic and the total (plastic + elastic) energies during nanoindentation, *U*_el_/*U*_tot_. These energies were calculated from the nanoindentation experiments as the areas between the unloading curve and the *x*-axis (*U*_el_) and between the loading curve and the *x*-axis (*U*_tot_). The elastic constants were determined using ultrasonic measurements (pulse-echo overlap technique) along with density assessment (Archimedes’ method).

### 3.4. Corrosion Characterization

Electrochemical polarization measurements were carried out using a PGSTAT120 Autolab potentiostat/galvanostat (Ecochemie). Prior to the measurements, Ti_40_Zr_20_Hf_20_Fe_20_ (atom %) disks from the rods were cut and carefully ground with emery paper (SiC) up to grit 4000 and further polished with 6, 3, and 1 μm diamond suspension. Subsequently, the samples were degreased in acetone and finally cleaned with distilled water. Electrochemical corrosion experiments were performed in naturally aerated simulated physiological Hank’s balanced salt solution (HBSS) at 37.5 °C. The composition of the HBSS solution was: 8.0 g·dm^−3^ NaCl + 0.4 g·dm^−3^ KCl + 0.04788 g·dm^−3^ Na_2_HPO_4_ + 0.06 g·dm^−3^ KH_2_PO_4_ + 0.185 g·dm^−3^ CaCl_2_·2H_2_O + 0.09767 g·dm^−3^ MgSO_4_ + 0.35 g·dm^−3^ NaHCO_3_ + 1.0 g·dm^−3^ D-glucose. A typical three-electrode cell configuration was used. The reference electrode consisted of a double junction Ag|AgCl electrode filled with 3M KCl inner solution and 1 M NaCl interchangeable outer solution (E(SHE)= +0.210 V). A platinum foil acted as the counter electrode. Before each polarization scan, the sample was immersed in the electrolyte for 30 min. Afterwards, the potential was swept toward the anodic direction starting from 500 mV below to the open-circuit potential (OCP) at a scan rate of 0.5 mV·s^−1^. The corrosion current density (*j*_corr_) values were determined using the Tafel extrapolation method [41].

The mechanical properties and the corrosion behaviour of Ti_40_Zr_20_Hf_20_Fe_20_ alloys were compared with those of commercial Ti–6Al–4V purchased from Goodfellow [42].

### 3.5. Biological Tests

#### 3.5.1. Cell Culture

MC3T3-E1 mouse preosteoblasts (ATCC) were cultured in α-minimum essential medium (MEMα, Invitrogen) with 10% foetal bovine serum (FBS, Gibco) in standard conditions (at 37 °C and 5% CO_2_). Alloy disks were glued individually onto a glass coverslip with silicone, introduced into a 4-multiwell culture plate and sterilized under UV light for at least 1 h. Once sterilized, 50,000 preosteoblasts were seeded into each well and cultured in standard conditions for 24 h. For all experiments three groups were analyzed: cells grown on top of the alloy, cells grown on the coverslip in the presence of the alloy and cells grown on the coverslip in absence of the alloy (control culture). All experiments were conducted in triplicate.

#### 3.5.2. Cell Viability Assay

Cytotoxicity was analysed by detecting intracellular esterases activity using the Live/Dead Viability/Cytotoxicity Kit for mammalian cells (Invitrogen), according to the manufacturer’s protocol. Images from different regions (alloy disk and coverslip) of each culture sample were captured using an Olympus IX71 inverted microscope equipped with epifluorescence. Data were analyzed for significance using the Fisher’s exact test for comparison between groups. Statistically significance was considered when *p* < 0.05.

#### 3.5.3. Scanning Electron Microscope (SEM) Analysis of Cells

Cells were rinsed twice in phosphate buffered saline (PBS), fixed in 4% paraformaldehyde in PBS for 45 min at room temperature (RT) and rinsed twice in PBS. Cell dehydratation was performed in a series of ethanol washes (50%, 70%, 90%, and twice 100%), 7 min each. Finally, samples were dried using hexamethyl disilazane (HMDS; Electron Microscopy Sciences) for 10 min. Samples were mounted on special stubs and analyzed using a SEM (Zeiss Merlin) equipped with energy-dispersive X-ray spectroscopy (EDX) analysis.

#### 3.5.4. Cell Adhesion Analysis

Cell adhesion onto the alloy was determined by the presence of focal contacts. Phalloidin was used to visualize stress fibers (actin filaments) whereas an antibody against vinculin was used to detect the focal contacts. Following the same protocol described for viability studies, 50,000 preosteoblasts were seeded into a well containing an alloy disk glued onto a coverslip. After 24 h of culture, cells were fixed in 4% paraformaldehyde (Sigma) in PBS for 45 min at RT, permeabilized with 0.1% Triton X-100 (Sigma) in PBS for 15 min and blocked for 25 min with 1% PBS-bovine serum albumin (BSA; Sigma) at RT. Samples were then incubated with a mouse anti-vinculin primary antibody (Chemicon) for 60 min at RT and washed with 1% PBS-BSA. Then, samples were incubated with a mixture of Alexa fluor 594-conjugated phalloidin (Invitrogen), Alexa fluor 488 goat anti-mouse IgG1, and Hoechst 33258 (both from Sigma) for 60 min at RT. Finally, samples were washed in 1% PBS-BSA and air dried. Samples were mounted on specific bottom glass dishes (MatTek) using Fluoroprep mounting solution (Biomerieux). Cells were imaged in a confocal laser scanning microscope (Leica SP5). Series of horizontal optical sections were collected at 400 nm intervals and projections were generated with Imaris software (Bitplane).

#### 3.5.5. Cell Differentiation Assay

Preosteoblasts differentiation in the presence of the alloy was analyzed through the detection of calcium deposits, a sign of ECM mineralization. To this aim, 500,000 preosteoblasts were seeded into 35 mm culture dishes containing an alloy disk glued to a coverslip with silicone. After 24 h in culture, MEMα medium was removed and differentiation medium, consisting of MEMα supplemented with 10–8 M dexamethasone, 50 µg/mL ascorbic acid and 8 mM β-glycerophosphate (all from Sigma), was added. Cells were cultured during 14 or 21 days in the presence of the differentiation medium, which was replaced every 3–4 days. Secreted calcium deposits were detected using Alizarin Red S staining. After 14 or 21 days in culture, cells were rinsed in PBS and fixed in 4% paraformaldehyde in PBS for 45 min at RT. Then, cells were washed twice with PBS and incubated with 2% Alizarin Red S (Sigma) for 30 min at RT. Finally, samples were washed with milliQ water and visualized using Olympus IX71 inverted microscope.

## 4. Conclusions

The synthesis and characterization of Ti_40_Zr_20_Hf_20_Fe_20_ alloy, in form of rods of 2 mm in diameter, by copper mold casting process is reported. Our results reveal that:

(i) The Ti_40_Zr_20_Hf_20_Fe_20_ rod is mechanically harder than Ti–6Al–4V and it shows lower Young’s modulus, as determined by nanoindentation tests.

(ii) The Ti_40_Zr_20_Hf_20_Fe_20_ alloy shows good corrosion resistance in HBSS, as evidenced by the passive behavior in a wide potential window and the low corrosion current densities observed. In comparison with commercial Ti–6Al–4V alloy, the Ti_40_Zr_20_Hf_20_Fe_20_ alloy presents slightly larger value of polarization resistance.

(iii) In terms of biocompatibility, the Ti_40_Zr_20_Hf_20_Fe_20_ alloy is not cytotoxic and preosteoblast cells can easily adhere onto its surface and differentiate into osteoblasts.

All abovementioned properties make the Ti_40_Zr_20_Hf_20_Fe_20_ alloy appealing as potential candidate to be used as biomaterial.
